# Supplementary material for: All-at-once RNA folding with 3D motif prediction framed by evolutionary information
Source: Nat Methods. 2025 Oct 3;22(10):2094–106. doi: 10.1038/s41592-025-02833-w (PMC12510878; doi:10.1038/s41592-025-02833-w)
Supplement: Supplementary file 3 — Source code to run CaCoFold-R3D. Data and results. [file 41592_2025_2833_MOESM3_ESM.gz › supplemental_material/results/CaCoFold-R3D/CaCoFold-R3D_Rfam/RF02283_TtnuCD6.R2R.sto.pdf]

RF02283_TtnuCD6

5′-U R Y ● G A U G A ● A ○ ● U ● C U U A G G A C G C C U U C U G A A A C G A Y U U U U C G U U U G U G A G A A Y A A ○ ● C ● R G ● A ● C U G A ● ●
